# Psychosocial Factors Affecting Community Integration in Schizophrenia Patients: The Moderating Role of Perceived Policy Effectiveness

**DOI:** 10.1002/brb3.71294

**Published:** 2026-04-21

**Authors:** Bora Coşar, Begüm Yalçın

**Affiliations:** ^1^ Department of Management and Organization, Logistics Program İstanbul Beykent University İstanbul Istanbul Türkiye; ^2^ Department of Management and Organization, Healthcare Management Program İstanbul Beykent University İstanbul Istanbul Türkiye

## Abstract

**Introduction:**

Schizophrenia patients in Istanbul, Türkiye, confront psychosocial and structural barriers to community integration, including pervasive stigma, self‐imposed social withdrawal, and limited trust in a fragmented mental health policy landscape. In this context, community integration refers to the acceptance of an individual as part of society, regardless of any illness or difference they may have, and their ability to participate equally in social, cultural, and economic life. The current structure and social context of mental health policies in Türkiye, in particular, are factors that significantly shape the motivation of individuals diagnosed with schizophrenia to participate in the process of integration into society. The purpose of this study is to investigate the impact of perceived discrimination, social withdrawal, and mental health self‐efficacy on integration into the community and to determine whether individuals’ perceptions of policy effectiveness moderate these relationships.

**Methods:**

In this cross‐sectional study, data were collected using a questionnaire from 427 schizophrenia patients receiving outpatient treatment at two public hospitals and seven community mental health centers in Istanbul, Türkiye. The questionnaire included socio‐demographic questions along with four scales: the Internalized Stigma of Mental Illness Scale, the General Mental Health Self‐Efficacy Scale, the Perceived Policy Effectiveness Scale, and the Community Integration Scale. The data obtained were analyzed using SPSS and SmartPLS software packages. Structural equation modelling‐based moderation analyses tested the direct effects of perceived stigma, social withdrawal, and self‐efficacy on community integration, as well as interaction terms involving policy perception.

**Results:**

Higher perceived discrimination (β = −0.42, *p* < 0.001) and greater social withdrawal (β = −0.37, *p* < 0.001) each significantly predicted lower community integration, while self‐efficacy emerged as a smaller positive effect (β = 0.36, *p* < 0.001). Moderation analyses revealed that positive policy perception had a limited buffering effect, reflecting patients’ persistent skepticism toward policy implementation. Specifically, even among those who viewed services as accessible, stigma and withdrawal undermined social participation.

**Conclusion:**

Findings underscore that, within Türkiye's current mental health system, strengthening individual agency through self‐efficacy alone is insufficient to overcome entrenched discrimination and isolation. Sustainable community integration requires structural change: policy reforms must extend beyond paper commitments to include adequately resourced services and long‐term anti‐stigma initiatives. Only by aligning legislative intent with real‐world service quality can policy perception become a genuine catalyst for integration.

**Patient or Public Contribution:**

During data collection, participants provided firsthand accounts of their experiences with stigma and service shortcomings. Their reflections on policy gaps and barriers to care informed the interpretation of findings and highlighted priorities for more responsive, patient‐centered mental health policy.

## Graphical abstract

This cross‐sectional study (*n* = 427, Istanbul) examines how perceived discrimination, social withdrawal, and mental‐health self‐efficacy shape community integration in schizophrenia. Discrimination and withdrawal lower integration, while self‐efficacy increases it. Perceived policy effectiveness shows small moderation across paths (*R* ^2^ = 0.48), insufficient to offset stigma‐related barriers.

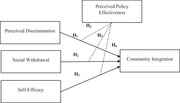

## 1 Introduction

Community integration, defined as the restoration of meaningful social roles, relationships, and participation in one's environment, including domains such as social participation, employment, and access to essential services, is a critical outcome in the recovery of individuals with schizophrenia (Pahwa et al. 2014). This concept is important because it improves long‐term functioning and quality of life by reducing the negative effects of psychosocial and structural factors experienced by individuals with schizophrenia (Ye et al. 2023). However, pervasive public stigma remains a formidable barrier: people with schizophrenia are frequently labeled as dangerous or unpredictable, prompting social distancing and exclusion in many societies, including Türkiye (Utz et al. 2019). Internalized stigma, wherein individuals apply negative societal attitudes to themselves, contributes to social withdrawal and reduced self‐esteem, further impairing social engagement and quality of life. However, some analytical evidence suggests that the relationship between internalized stigma and social withdrawal is not one‐sided. It is emphasized that social withdrawal and loneliness also reinforce the individual's negative self‐image, thereby deepening the internalization of stigma (Turan et al. 2016). At the same time, this interaction can be strengthened or weakened depending on factors such as the individual's level of access to services and the cultural norms in which they live (Ahad et al. 2023). Some meta‐analytic evidence indicates that internalized stigma is strongly linked to decreased self‐efficacy and functional outcomes in schizophrenia populations worldwide (Jahn et al. 2020).

Self‐efficacy, the belief in one's ability to manage illness‐related challenges, is a well‐established predictor of better psychosocial functioning and community participation among individuals with serious mental illness (Kurtz et al. 2013). Studies have shown that higher self‐efficacy mitigates the impact of negative symptoms on daily functioning, provided that patients possess adequate illness insight. Conversely, social withdrawal, often a self‐protective response to discrimination, exacerbates loneliness and undermines opportunities for meaningful engagement, leading to a vicious cycle of isolation and symptom exacerbation (Fulford and Holt 2023). In this context, self‐efficacy and social withdrawal are also interrelated. Studies indicate that high self‐efficacy decreases social withdrawal because it supports social participation and functioning; conversely, low self‐efficacy may perpetuate social withdrawal due to negative self‐evaluation (Larasati Yunanto et al. 2021; Vinu and Georgiades 2025).

Türkiye's mental health policy framework formally emphasizes decentralization and community‐based care, as outlined in the 2006 Mental Health Policy and subsequent action plans up to 2023. Yet these reforms have largely remained on paper: no standalone Mental Health Law was enacted, and resources allocated to community mental health centers (CMHCs) are insufficient to meet demand (Bilir and Artvinli 2021; WHO 2009). This limited application of policy offers a critical context for comprehending the community integration obstacles examined in this study. Policy interventions and inclusive social arrangements that support individuals diagnosed with schizophrenia may have the potential to mitigate the effects of internalized stigma and social withdrawal. In this regard, perceived policy effectiveness may play a moderating role in the impact of the relationship between internalized stigma, social withdrawal, and self‐efficacy on community integration.

Consequently, patients’ perceptions of policy effectiveness are often marked by skepticism. Qualitative inquiries describe Turkish schizophrenia patients viewing services as fragmented, bureaucratic, and poorly coordinated, which reduces trust and adherence to psychosocial programs (Aktaş and Ayhan 2025). Even when EU or WHO projects temporarily bolster CMHC capacity, such as the EU‐financed SIHHAT initiative, sustainability concerns lead patients to question the long‐term reliability of services.

This study covers 427 schizophrenia patients receiving outpatient treatment at secondary and tertiary healthcare institutions in Istanbul, Türkiye, and aims to examine the psychosocial factors affecting patients’ social integration. The study evaluated the direct effects of perceived discrimination, social withdrawal, and self‐efficacy regarding mental health on social integration; additionally, the moderating role of perceived policy effectiveness in these relationships was tested. This study examines the psychosocial factors that influence the degree to which schizophrenia patients are well integrated into society, particularly in light of the Turkish healthcare system and social policies. The results provide a unique contribution to both national policy development and the design of patient‐centered interventions.

By combining quantitative evaluations with a critical analysis of Türkiye's policy environment, the study seeks to reveal the connections between individual and structural factors that affect recovery pathways. This study addresses a gap in the literature by examining a novel moderation hypothesis: that policy perceptions may lessen the detrimental effects of stigma and withdrawal on integration outcomes. This strategy provides both theoretical progress and practical recommendations for policy reform in Türkiye's changing mental health framework.

### 1.1 Theoretical Framework and Literature Review

Internalized stigma is recognized as a significant barrier to psychosocial recovery in schizophrenia, occurring when patients turn societal prejudice inward. Numerous studies across Europe report that many people with schizophrenia face discrimination in employment, housing, and social roles (Lasalvia et al. 2021). Considering the body of research, social disengagement is a defensive tactic against rejection that is triggered by perceived discrimination and self‐worth. Research shows that, independent of medication adherence, higher initial stigma predicts greater isolation and worsening of symptoms at 6 months. (Prizeman et al. 2025). These findings support the proposed causal model by strengthening the conceptual link between internalized stigma and perceived discrimination, and social withdrawal. According to urban surveys conducted in Türkiye, more than 70% of the general public has distancing attitudes toward schizophrenia, which exacerbates patients’ self‐stigma and causes them to hide their symptoms and avoid social situations (Kılıç‐Demir and Kızılpınar 2024). Collectively, these findings underscore that perceived discrimination and subsequent social withdrawal constitute a self‐reinforcing cycle of exclusion, evidenced across diverse contexts (Fulford and Holt 2023). Based on the literature, the following hypotheses are proposed. **H_1_**: Perceived discrimination has a negative impact on community integration. **H_2_**: Social withdrawal has a negative impact on community integration.

One important factor influencing functional outcomes in schizophrenia is self‐efficacy, or the belief in one's ability to manage symptoms and day‐to‐day difficulties (Zhang et al. 2024). Cognitive‐behavioral programs focusing on self‐efficacy in patients with chronic schizophrenia appear to produce modest improvements in community participation and occupational engagement (Zheng et al. 2023). After adjusting for symptom severity and support network size, initial self‐efficacy appears to predict quality of life several years later. Interventions that increase self‐efficacy, such as peer‐led support groups, are associated with significantly improved medication adherence and social activity levels, according to psychiatric reviews (Sun et al. 2022). Turkish pilot projects embedding self‑efficacy training in community mental health centers have replicated these effects, reporting significant gains in patient autonomy and social contacts despite the presence of structural barriers (Bilkay et al. 2024). The following hypothesis is proposed to address this objective. **H_3_**: Mental health self‐efficacy has a positive impact on community integration.

Originally stated in the 2006 Mental Health Policy and further supported by action plans through 2023, Türkiye's official mental health framework places a strong emphasis on deinstitutionalization and community‐based care, but there are serious shortcomings in the implementation of these objectives. The policy on paper has few enforcement tools because no stand‐alone mental health law has been passed, even though EU‐funded initiatives temporarily expanded community services (Artvinli and Uslu 2023). Patients perceive current programs as disjointed and under‐resourced, which lowers trust and adherence to psychosocial interventions, according to a recent qualitative study of service users with schizophrenia (Aktaş and Ayhan 2025). WHO data confirms systemic deficiencies: Türkiye lacks a dedicated budget line for mental health, which leads to chronic understaffing of community mental health centers, and employs only 1.64 psychiatrists per 100,000 population, which is less than the 5.0 average in high‐income countries. Türkiye also lags behind neighboring countries such as Greece (3.1) and Bulgaria (2.8) in terms of the number of psychiatrists per capita (World Health Organization 2018). Scholarly critiques emphasize that policy rhetoric has not translated into sustainable workforce development or service coordination, so patients continue to experience bureaucratic obstacles when seeking outpatient support (Yilmaz and Bilir 2022). Consequently, the policy environment for individuals with schizophrenia remains characterized by implementation gaps and limited patient engagement, impeding the intended shift toward holistic, community‑based recovery (Ayhan et al. 2025).

Community integration, measured by social network breadth, employment, and leisure engagement, is a key recovery metric in schizophrenia research. Psychosocial treatments indicate that assertive community treatment, supported employment, and social skills training yield small to moderate improvements in integration outcomes, influenced by funding stability and staff ratios. Personalized community programs increase social participation by 40% and reduce hospitalization rates by 25% at six‐month follow‐up, according to a meta‐analysis of low‐ and middle‐income countries (Asher et al. 2017). Evaluations of CMHC pilots in Türkiye show modest increases in patient autonomy and participation in leisure activities; however, the long‐term effects are limited by irregular hours and staff turnover. These collective findings highlight that while structured interventions can enhance integration, resource constraints and policy shortfalls in Türkiye curtail their scalability and sustainability (Kohrt et al. 2018). Consequently, this study aims to investigate whether perceived policy effectiveness serves as a moderating factor in the relationships between psychosocial factors such as perceived discrimination, social withdrawal, and self‐efficacy regarding mental health and community integration. Hypotheses 4, 5, and 6 have been formulated to address this inquiry. **H_4_**: Perceived policy effectiveness has a moderating effect on the relationship between perceived discrimination and community integration. **H_5_**: Perceived policy effectiveness has a moderating effect on the relationship between social withdrawal and community integration. **H_6_**: Perceived policy effectiveness has a moderating effect on the relationship between self‐efficacy and community integration.

The importance of this study is reflected in its potential to create a model that clarifies the effects of psychosocial factors such as perceived discrimination, social withdrawal, and self‐efficacy regarding mental health on community integration, as well as the moderating role of perceived policy effectiveness in these effects. While existing studies have explored the relationships between these variables independently, the absence of research that combines these relationships into a single model highlights the critical gap that this study aims to address.

## 2 Methods

### 2.1 Study Design

This study, conducted at two public hospitals and seven community mental health centers in Istanbul, Türkiye, is a cross‐sectional study designed within the framework of a quantitative research approach. The study was designed to identify psychosocial factors affecting the community integration of individuals diagnosed with schizophrenia and to test the moderating role of perceived policy effectiveness in the relationships between these factors. In this context, perceived discrimination, social withdrawal, and self‐efficacy related to mental health were considered exogenous variables, while community integration was considered the endogenous variable. In addition, interaction terms between perceived policy effectiveness and each exogenous variable were specified to test moderation effects. The cross‐sectional nature of the study allowed data to be collected at a single point in time and enabled the relationships between variables to be evaluated at a correlational rather than causal level.

### 2.2 Sample and Procedure

The study population consists of individuals diagnosed with schizophrenia who are receiving outpatient treatment at two hospitals affiliated with the Ministry of Health and seven community mental health centers in Istanbul, Türkiye. The primary factor in selecting individuals diagnosed with schizophrenia is that this group is among the most frequently stigmatized, discriminated against, and socially excluded segments of society, and their levels of community integration are generally low.

In the sample selection, the hospitals and community mental health centers where the research was conducted were treated as natural clusters representing the geographical and institutional diversity of the population. Patients were assessed based on accessibility and voluntariness during the data collection process, and data were collected accordingly.

No clear data were obtained on the number of schizophrenia patients receiving outpatient treatment at hospitals and community mental health centers during the data collection period (September 2024‐March 2025). Therefore, in calculating the sample size, the sample size corresponding to a population size of 100,000 (384) was considered for cases where the population was unknown (Ural and Kılıç 2005). In this study, data were collected from 427 patients using convenience sampling.

In the study, questions were asked about gender, age, marital status, educational status, employment status, living arrangements, and income level in order to determine the socio‐demographic characteristics of the participants. Of the 427 participants in the study, 64.2% were male, and 71.9% were married. Looking at the age distribution, the highest participation rate was in the 25–44 age group (54.1%). This indicates that the majority of participants were of working age. The percentage of individuals aged 55 and over was relatively low at 8.9%. In terms of educational level, the highest percentage was among primary school graduates (43.6%). This is followed by high school graduates (40.8%), university graduates (12.5%), and postgraduate degree holders (3.1%). In terms of employment status, more than half of the participants are unemployed (58.3%), and only 12.6% have full‐time jobs. The proportion of those working in irregular/temporary jobs is 15.5%, while retirees account for 8.9% and students for 4.7%. In terms of living arrangements, the majority of participants live with their families (68.1%), and 64.2% belong to the low‐income group.

### 2.3 Inclusion and Exclusion Criteria

Inclusion criteria for the study: (1) Having been diagnosed with schizophrenia by a psychiatrist, (2) Receiving outpatient treatment at a secondary or tertiary healthcare facility designated in Istanbul, Türkiye, (3) Being over 18 years of age, (4) Being able to give informed consent and complete self‐assessment questionnaires, (5) Having the cognitive and social competence to answer the questions on the questionnaire form. Assessment of cognitive and communicative impairment relied on clinicians’ judgment of participants’ capacity to comprehend and complete the questionnaire, without the use of a formal cognitive screening instrument.

The exclusion criteria are defined as (1) having serious neurological disorders or psychiatric disorders that could significantly impair cognitive and social functioning, (2) experiencing an episode or being hospitalized during data collection, (3) being unable to give informed consent or refusing to do so, and (4) inability to answer the questions in the questionnaire due to cognitive and communicative impairment.

### 2.4 Measures

In the study, a total of four different scales were used, remaining faithful to their original forms, and the questionnaire consists of 38 items. In addition, seven questions aimed at determining the socio‐demographic characteristics of the participants were included in the questionnaire. Detailed information about the scales is as follows:

Perceived discrimination and social withdrawal were assessed using the *Internalized Stigma of Mental Illness Scale (ISMI)* developed by Ritsher, Otilingam, and Grajales (2003), which has demonstrated strong reliability and validity in schizophrenia samples (Ritsher et al. 2003). The Turkish adaptation was done by Ersoy and Varan (2007). The perceived discrimination dimension of the scale consists of 5 items, while the social withdrawal dimension consists of 6 items. The scale items are four‐point Likert‐type (1 = Strongly disagree, 4 = Strongly agree). As a result of the reliability analyses of the scale, the total Cronbach's Alpha coefficient was reported as 0.90.

Mental health self‐efficacy was assessed using a measurement tool developed by Frank, Lee, Fikretoglu, and Bailey (2021) and called the *General Mental Health Self‐Efficacy Scale* (Frank et al. 2021). The scale items were translated by bilingual researchers, and exploratory factor analysis was conducted prior to hypothesis testing. All items loaded on the expected factor structure, supporting the use of the original scale in the present Turkish sample. The scale consists of 14 items and a single sub‐dimension. The scale items are five‐point Likert‐type (1 = Strongly disagree, 5 = Strongly agree). As a result of the scale's reliability analyses, the total Cronbach's Alpha coefficient was reported as 0.88.

Perceived policy effectiveness was adapted based on the material *“Mental Health Policy, Plans, and Programs”* based on the World Health Organization's publication Guidance on Mental Health Policy and Strategic Action Plans: Module 3 (2025) (World Health Organization 2025). The relevant scale consists of three items and a single sub‐dimension. The scale items are 5‐point Likert‐type (1 = Strongly disagree, 5 = Strongly agree).

Community integration was assessed using the *Community Integration Measure (CIM)* developed by McColl, Davies, Carlson, Johnston, and Minnes (2001) (McColl et al. 2001). The Turkish adaptation was done by Bekiroğlu and Yılmaz (2021). The scale consists of 10 items and a single sub‐dimension. The scale items are 5‐point Likert‐type (1 = Strongly disagree, 5 = Strongly agree). As a result of the scale's reliability analyses, the total Cronbach's Alpha coefficient was reported as 0.87.

### 2.5 Data Analysis

Statistical analyses of the data obtained in this study were performed using SPSS and SmartPLS software packages. The analysis process began with calculating the reliability levels of the scales used and obtaining basic descriptive statistics. Correlation analysis was used to reveal the direction and strength of the relationships between variables. Subsequently, the fit indices of the conceptual model were evaluated. These indices, calculated within the scope of Structural Equation Modeling (SEM), were used to determine how well the model fit the observed data. Finally, a path analysis of the conceptual model was performed. This analysis examined the direct effects of exogenous variables on the endogenous variable, as well as the moderating role of perceived policy effectiveness, in the model.

### 2.6 Ethical Considerations

Ethical approval for this study was obtained from the Ethics Committee for Social and Human Sciences at a foundation university in Türkiye. Additionally, they were obtained from the public hospitals and community mental health centers where the study was conducted. Participants were informed about the purpose of the study prior to the survey, and participation was voluntary. The Declaration of Helsinki was applied throughout all stages of the study.

## 3 Results

Before proceeding to test the research model and hypotheses, a correlation analysis was conducted to obtain preliminary information about the direction and strength of the relationships between the variables. Along with this analysis, the reliability coefficients for the variables used in the study are also presented in Table 1.

**TABLE 1:** Reliability and correlation analysis results of the conceptual model.

Construct	α	CR	AVE	1	2	3	4	5
1. Perceived discrimination	0.89	0.91	0.68	1.00	0.56	−0.29	−0.32	−0.26
2. Social withdrawal	0.87	0.89	0.65		1.00	−0.37	−0.23	−0.22
3. Self‐Efficacy	0.85	0.88	0.62			1.00	0.44	0.51
4. Policy perception	0.82	0.86	0.58				1.00	0.39
5. Community integration	0.90	0.92	0.70					1.00

According to Table 1, Cronbach's α and composite reliability values exceeding the 0.70 threshold indicate that each construct exhibits adequate internal consistency and internal consistency reliability within the PLS SEM framework. Average Variance Extracted (AVE) values above 0.50 demonstrate that each latent variable explains more than half of the variance in its indicators, supporting convergent validity). The fact that the square roots of AVE (diagonal entries) exceed the interconstruct correlations confirms that each construct is more strongly associated with its items than with other constructs, laying the groundwork for discriminant validity (Cheung et al. 2024). The study's scales’ structural validity was examined using Confirmatory Factor Analysis (CFA). Table 2 provides a detailed presentation of the fit indices that were determined to be statistically significant in the CFA results.

**TABLE 2:** Summary of fit indices of the conceptual model.

Fit index	Value	Threshold
SRMR	0.062	< 0.08
NFI	0.91	> 0.90
*χ* ^2^/df	1.85	< 3.00
*R* ^2^ (Community integration)	0.48	—

According to Table 2, the Standardized Root Mean Square Residual (SRMR) value of 0.062 is below the recommended cutoff of 0.08, indicating a good model fit. An NFI of 0.91 further supports model adequacy (threshold >  0.90), and a *χ* ^2^/df ratio of 1.85 (below 3.00) suggests that the model does not overfit the data (Cheung et al. 2024).

**FIGURE 1:**
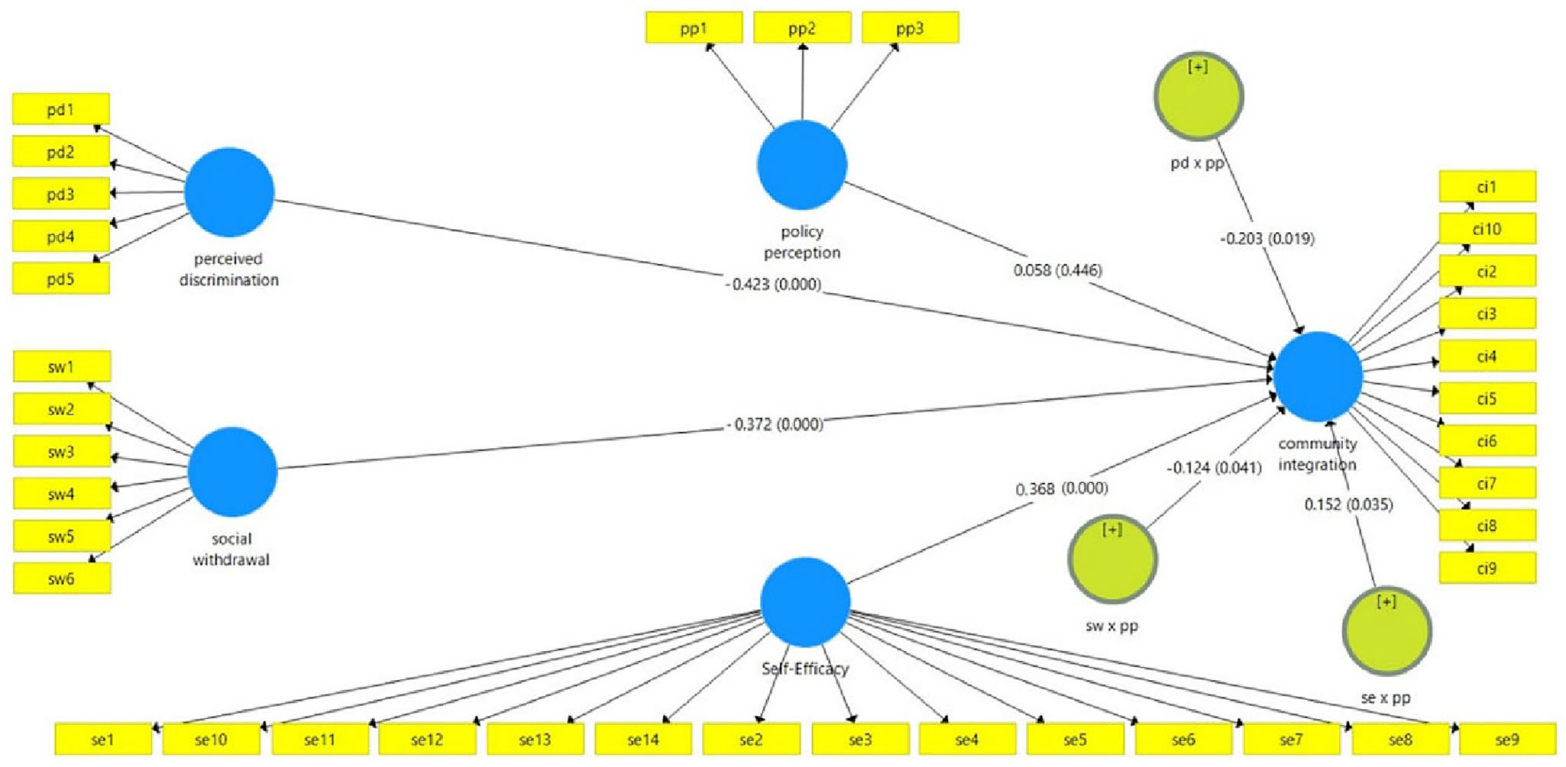
The moderating role of perceived policy effectiveness in the impact of psychosocial factors on community integration.

Structural Equation Modeling (SEM) was used to test the hypotheses included in the proposed research model. In this regard, a path analysis model was created regarding the psychosocial factors affecting the community integration of individuals diagnosed with schizophrenia and the moderating role of perceived policy effectiveness in the relationships between these factors. The results of the structural model established in the study and forming the main theme of the work are presented in Figure 1.

The hypotheses contained in the suggested research model were tested using structural equation modeling, or SEM. Accordingly, a path analysis model specifying exogenous variables, an endogenous outcome, and interaction terms was developed. Table 3 displays the results of the study's structural model, which serves as the work's central focus.

**TABLE 3:** Path analysis of the conceptual model.

Path	β	*t*‐value	*p*‐value
Perceived discrimination → Community integration	−0.42	7.12	< 0.001
Social withdrawal → Community integration	−0.37	6.45	< 0.001
Self‐Efficacy → Community integration	0.36	5.98	< 0.001
Discrimination × Policy perception → Community integration	−0.20	2.35	0.019
Self‐efficacy × Policy perception → Community integration	0.15	2.11	0.035
Social withdrawal × Policy perception → Community integration	−0.12	2.05	0.041

Path coefficients (β) represent the strength and direction of hypothesized relationships, and their statistical significance is assessed via bootstrapping (e.g., 5000 resamples) in SmartPLS 4. According to Table 3, all direct effects and interaction terms in the model reached significance at *p* < 0.05, confirming that perceived discrimination and social withdrawal negatively impact community integration, while self‐efficacy and policy perception exert positive and buffering influences. Within this framework, the research hypotheses H_1_ (Perceived discrimination has a negative impact on community integration), H_2_ (Social withdrawal has a negative impact on community integration), and H_3_ (Mental health self‐efficacy has a positive impact on community integration) were accepted.

The *f* ^2^ effect size metric quantifies each predictor's substantive contribution to explained variance, with conventions of 0.00 ≤ ^2^ < 0.15 being small, 0.15 ≤ *f* ^2^ < 0.35 being moderate, and *f* ^2^ ≥ 0.35 being large. According to Table 4, medium effect sizes for perceived discrimination (*f* ^2^ = 0.26) and social withdrawal (*f* ^2^ = 0.22) highlight their practical importance, whereas the moderating roles of policy perception yield small but meaningful buffering effects (*f* ^2^ = 0.05–0.11). Within this framework, the research hypotheses H_4_ (Perceived policy effectiveness has a moderating effect on the relationship between perceived discrimination and community integration), H_5_ (Perceived policy effectiveness has a moderating effect on the relationship between social withdrawal and community integration), and H_6_ (Perceived policy effectiveness has a moderating effect on the relationship between self‐efficacy and community integration) were accepted. The findings indicate that current policy regulations are insufficient to fully improve patients’ social participation and that more concrete and confidence‐building measures are needed.

**TABLE 4:** Model pathways and effect levels.

Path	*f* ^2^	Effect level
Perceived Discrimination → Community Integration	0.26	Medium
Social Withdrawal → Community Integration	0.22	Medium
Self‐Efficacy → Community Integration	0.19	Medium
Policy × Discrimination (moderation)	0.11	Small
Policy × Self‐Efficacy (moderation)	0.08	Small
Policy × Social Withdrawal (moderation)	0.05	Small

A simple slopes analysis was conducted to further illustrate the moderating role of policy perception. The following interaction plots clarify how different levels of policy perception (standardized at +1 SD and −1 SD) alter the impact of psychosocial factors on community integration.

**FIGURE 2:**
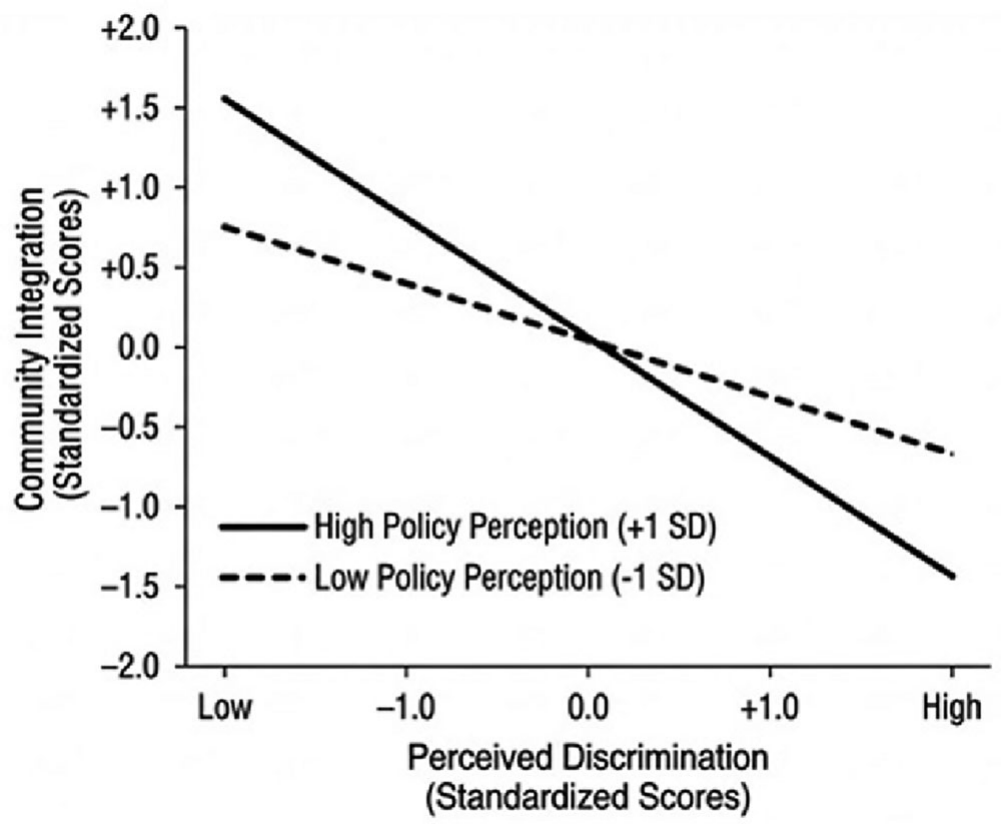
Moderating effect of perceived policy effectiveness on the association between perceived discrimination and community integration (simple slopes at ±1 SD).

Simple slopes analysis indicated that perceived discrimination was negatively associated with community integration at both levels of perceived policy effectiveness. As shown in Figure 2, this negative association was stronger when perceived policy effectiveness was high (+1 SD; β = −0.62, *p* < 0.01) than when it was low (−1 SD; β = −0.22, *p* < 0.05), indicating a strengthening (rather than buffering) moderation pattern.

**FIGURE 3:**
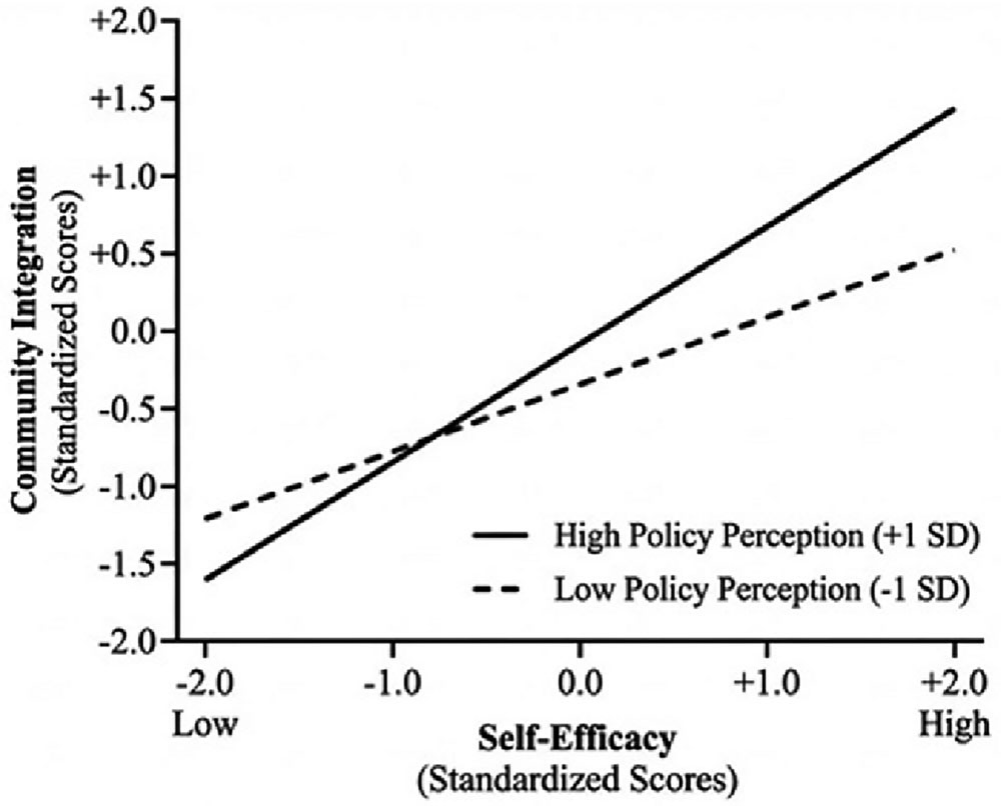
Moderating effect of perceived policy effectiveness on the association between self‐efficacy and community integration (simple slopes at ±1 SD).

Simple slopes analysis indicated that self‐efficacy was positively associated with community integration at both levels of perceived policy effectiveness. As shown in Figure 3, this positive association was significantly stronger at high levels of perceived policy effectiveness (+1 SD; β = 0.51, *p* < 0.01) compared to low levels (−1 SD; β = 0.21, *p* < 0.05), suggesting that favorable policy perceptions amplify the beneficial role of self‐efficacy in community integration.

**FIGURE 4:**
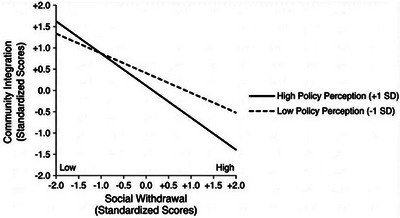
Moderating effect of perceived policy effectiveness on the association between social withdrawal and community integration (simple slopes at ±1 SD).

Simple slopes analysis indicated that social withdrawal was negatively associated with community integration at both levels of perceived policy effectiveness. As shown in Figure 4, this negative association was stronger at high levels of perceived policy effectiveness (+1 SD; β = −0.49, *p* < 0.01) than at low levels (−1 SD; β = −0.25, *p* < 0.05), indicating a strengthening moderation effect.

## 4 Discussion

This study examined how perceived discrimination, social withdrawal, and mental health self‐efficacy are related to community integration among schizophrenia patients in Türkiye, and how perceived policy effectiveness influences these relationships. Overall, the findings indicate that discrimination (β = −0.42, *p* < 0.001) and social withdrawal (β = −0.37, *p* < 0.001) had moderate negative effects on community integration, while self‐efficacy (β = 0.36, *p* < 0.001) made a positive contribution, albeit to a lesser extent. Importantly, perceived policy effectiveness did not function as a uniform protective factor; instead, its regulatory role varied depending on the psychosocial context.

Consistent with previous international research, higher levels of perceived discrimination were associated with significantly lower community integration (Hack et al. 2020). This finding aligns with extensive evidence showing that stigma weakens social participation and reinforces exclusion among individuals with severe mental illness (Ahad et al. 2023; Von Lersner et al. 2019). The moderate effect size observed in this study emphasizes that discrimination remains a fundamental barrier to integration, even in systems formally oriented toward community‐based care. Similar patterns have been documented in Western and non‐Western contexts, demonstrating that stigma functions as a persistent structural and interpersonal constraint on recovery (Sum et al. 2024; Pattyn et al. 2014; Özdemir and Aras 2023; Corrigan et al. 2012).

Social withdrawal, showing a moderately negative relationship with community integration, reinforced its role as one of the fundamental mechanisms of social isolation in schizophrenia. Withdrawal limits opportunities for community interaction and skill acquisition, thereby restricting participation in social life. The magnitude and direction of this relationship are consistent with previous findings linking negative symptoms and avoidance behaviors to long‐term functional impairment (Fulford and Holt 2023). In contrast, mental health self‐efficacy showed a positive relationship with community integration, supporting recovery‐focused models that emphasize personal competence and confidence in managing illness‐related challenges. Although the effect size was smaller than that for discrimination or withdrawal, self‐efficacy still made a meaningful contribution to integration outcomes; this is consistent with evidence linking higher self‐efficacy to improved functioning and quality of life (Mancini 2007; Pratt et al. 2005).

Importantly, simple slope analyses revealed that perceived policy effectiveness did not consistently buffer negative psychosocial effects. Positive policy perceptions strengthened the positive relationship between self‐efficacy and social integration while also intensifying the negative effects of discrimination and social withdrawal. This pattern indicates that policy perception operates conditionally and contextually. When individuals have high expectations regarding policy effectiveness, persistent experiences of stigmatization or disconnection can increase disappointment and hopelessness, thereby reinforcing negative effects on integration. Conversely, under low policy perceptions, expectations may already be constrained, leading to relatively weak relationships (Uddin and Adhikari 2024).

When taken together, these findings suggest that perceived policy effectiveness alone is not sufficient to compensate for entrenched psychosocial barriers. Rather than serving as a universal protective buffer, policy perception interacts with lived social experiences and, when expectations are not met, sometimes appears to exacerbate disadvantage. This nuanced moderation pattern highlights the importance of aligning policy commitments with concrete, visible improvements in community‐based services for policy credibility to translate into meaningful gains in community integration.

### 4.1 Practical Implications

These shortcomings have clear implications. The prominence of stigma and withdrawal in our findings reinforces that addressing social attitudes must be central to integration efforts [64]. In other countries, anti‐stigma initiatives (mass media, contact programs) have produced modest gains in public attitudes (Chisholm et al. 2016). Türkiye could similarly invest in long‐term community interventions to counter stigma (Corrigan et al. 2012; Wainberg et al. 2017). Meanwhile, interventions to bolster patient self‐efficacy are warranted (Topuzoglu 2021). Psychosocial rehabilitation and peer‐support programs can help patients rebuild confidence and social skills, countering defeatist beliefs and learned helplessness (as theorized by relapse research) (Pratt et al. 2005). For example, cognitive therapies that target “defeatist attitudes” have been shown to reduce withdrawal and improve function. Empowerment initiatives—from supported employment to peer‐led groups—may likewise translate the abstract goal of self‐efficacy into concrete progress (Barlati et al. 2024).

At the system level, our results underscore urgent needs in Türkiye's mental health policy. Simply drafting new documents will not suffice: robust funding and enforcement are required (Bond et al. 2016). Expanding CMHCs and integrating them with primary care (a shortfall noted by Turkish GPs) should be a priority (Kisa and Younis 2006). As family physicians themselves suggest, mandatory mental health training and better referral links to CMHCs could greatly improve community care. In addition, legal reforms are needed. Parliament should finalize a mental health law that clarifies patients’ rights, involuntary admission criteria, and standards of care. Such a law would provide a framework to hold services accountable (Sharifi et al. 2021). Civil society should also be empowered: patient and family organizations must be invited to shape policy and service design, as recovery‐oriented practice requires.

### 4.2 Strengths, Limitations, and Future Directions

This study is considered to have significantly advanced the literature by comprehensively addressing the psychosocial factors (perceived discrimination, social withdrawal, and self‐efficacy) that affect the community integration of individuals diagnosed with schizophrenia and by including perceived policy effectiveness as a moderator variable in the model. The study's strengths include the use of a relatively large sample of schizophrenia patients receiving outpatient treatment at two public hospitals and seven community mental health centers (*n* = 427), the adoption of contemporary research approaches (PLS‐SEM), and the evaluation of original policy data, particularly in the Turkish context.

In addition to the important findings obtained in the study, some limitations have emerged. First, the adoption of a cross‐sectional design in the research has been limited in its ability to determine causal relationships between variables. Longitudinal studies are needed to more clearly identify changes over time in the psychosocial factors affecting the community integration of schizophrenia patients.

Second, although the study was conducted voluntarily, measurement limitations may arise due to methodological errors such as social desirability bias and recall bias, as self‐report scales were used in the study. Therefore, it is recommended that qualitative data support future studies.

Third, participants were selected only from public hospitals and community mental health centers in Istanbul, Türkiye. This may limit the generalizability of the findings to different regions and heterogeneous cultures. It is recommended that future studies be replicated in broader or different cultural contexts.

Fourth, several clinical characteristics known to influence psychosocial functioning in schizophrenia (such as duration of illness, symptom severity, age at diagnosis, and treatment adherence) were not assessed in the present study. The absence of these variables limits the ability to account for their potential confounding effects on community integration. Future research would benefit from incorporating these clinical indicators to better disentangle the interplay between psychosocial factors, policy‐related perceptions, and functional outcomes. Additionally, because cognitive functioning was not assessed using a standardized instrument, exclusion based on cognitive or communicative impairment relied on clinical judgment, which may have introduced variability in participant selection.

Finally, considering that the buffering effect of policy perception is limited, experimental studies and longitudinal follow‐up studies evaluating the impact of concrete policy interventions in future studies are expected to make significant contributions to the literature.

## 5 Conclusion

This study examined the impact of psychosocial factors such as perceived discrimination, social withdrawal, and mental health self‐efficacy on community integration and revealed the moderating role of perceived policy effectiveness in these relationships. The findings show that high levels of perceived discrimination and social withdrawal negatively affect community integration in individuals diagnosed with schizophrenia, whereas high self‐efficacy supports community integration. Furthermore, it was found that policy perception has a small but statistically significant effect on these relationships and that perceived policy effectiveness plays a moderator role in the impact of these psychosocial factors on community integration. However, positive perceptions of mental health policy proved insufficient to act against deep‐rooted discrimination and isolation, underscoring a critical gap between policy intent and real‐world impact. To bridge this divide, Türkiye must pair legislative reform with sustained investment in community‑based services, workforce development, and anti‑stigma initiatives. Empowering patients through targeted psychosocial interventions, such as peer support and cognitive‑behavioral programs, can bolster self‑efficacy and foster social engagement. Ultimately, only a holistic strategy that aligns robust policy frameworks with practical, patient‐centered supports will transform mental health care from rhetoric into tangible community integration.

## Author Contributions

Conceptualization: B.C., B.Y.; Writing – original draft: B.C., B.Y.; Methodology: B.C., B.Y.; Validation: B.C.; Writing – review and editing: B.C., B.Y.; Data curation: B.C.; Resources: B.C., B.Y.

## Funding

The authors have nothing to report.

## Data Availability

The data obtained in this study are not publicly available due to the sensitive health information they contain regarding schizophrenia patients. However, subject to all ethical and legal requirements, the corresponding author may provide anonymized data supporting the findings of the study upon reasonable request.
